# Characterization of a novel VenusX orthogonal dual‐layer multileaf collimator

**DOI:** 10.1002/acm2.14357

**Published:** 2024-04-15

**Authors:** Qingxin Wang, Qifeng Li, Zhongqiu Wang, Chengwen Yang, Daguang Zhang, Jun Wang, Ping Wang, Wei Wang

**Affiliations:** ^1^ School of Precision Instrument and Opto‐Electronics Engineering Tianjin University Tianjin China; ^2^ Department of Radiation Oncology Tianjin Medical University Cancer Institute & Hospital National Clinical Research Center for Cancer Tianjin's Clinical Research Center for Cancer Key Laboratory of Cancer Prevention and Therapy Tianjin China; ^3^ Department of Radiation Oncology Tianjin Cancer Hospital Airport Hospital Tianjin China

**Keywords:** LinaTech VenusX linear accelerator, multileaf collimator, orthogonal dual‐layer MLC, radiotherapy

## Abstract

**Purpose:**

To investigate and characterize the performance of a novel orthogonal dual‐layer alpha multileaf collimator (αMLC) mounted on the LinaTech VenusX linac.

**Methods:**

We evaluated leaf positioning accuracy and reproducibility using an electronic portal imaging device through the picket fence test. The average, interleaf, intraleaf, and leaf tip transmissions of the single and dual layers were measured using an ionization chamber. Square and rhombus fields were used to evaluate the leaf penumbra of αMLC. To investigate the advantages of the orthogonal dual‐layer multileaf collimator (MLC) in field shaping, right triangular and circular pattern fields were formed using both the dual layers and single layers of the αMLC.

**Results:**

The average maximum positioning deviations of the upper and lower αMLC over 1 year were 0.76 ± 0.09 mm and 0.62 ± 0.07 mm, respectively. The average transmissions were 1.87%, 1.83%, and 0.03% for the upper‐, lower‐ and dual‐layer αMLC, respectively. The maximum interleaf transmissions of the lower‐ and dual‐layer were 2.43% and 0.17%, respectively. The leaf tip transmissions were 9.34% and 0.25%, respectively. The penumbra of the square field was 6.2 mm in the X direction and 8.0 mm in the Y direction. The average penumbras of the rhombus fields with side lengths of 5 and 10 cm were 3.6 and 4.9 mm, respectively. For the right triangular and circular fields, the fields shaped by the dual‐layer leaves were much closer to the set field than those shaped by single‐layer leaves. The dose undulation amplitude of the 50% isodose lines and leaf stepping angle change of the dual‐layer leaves were smaller than those of the single‐layer leaves.

**Conclusions:**

The αMLC benefits from its orthogonal dual‐layer design. Leaf transmission, dose undulations at the field edge, and MLC field dependence of the leaf stepping angle of the dual‐layer αMLC were remarkably reduced.

## 1 INTRODUCTION

The multileaf collimator (MLC) is the most widely used tool for modulating beam fluence in intensity‐modulated radiotherapy (IMRT) and volumetric modulated arc therapy (VMAT).^1^, ^2^, ^3^, ^4^, ^5^ MLC has been used in radiotherapy for over 30 years,^6^, ^7^ and its design characteristics influence treatment plan dose distributions.^8^, ^9^, ^10^, ^11^, ^12^, ^13^, ^14^ Single‐layer MLC architecture is primarily used in medical linear accelerators, such as the Varian Millennium 120‐leaf MLC system^15^, ^16^ and the Agility collimator of the Elekta MLC system.^6^

Unfortunately, the application of single‐layer MLC in radiotherapy has many limitations. Radiation leakage increases the dose both inside and outside the target volume, which is undesirable.^17^ Additionally, the projection width of the traditional single‐layer MLC on the isocenter plane typically ranges from 0.25 to 1.0 cm. Consequently, the stepped boundary of the irradiation field makes it challenging to achieve a smooth target boundary.^18^, ^19^ Generally, a good MLC should possess high leaf positioning accuracy, low leaf transmission, small penumbra, and the ability to accurately create various clinically required complex fields.^6^, ^10^, ^17^, ^20^

VenusX is a medical linear accelerator (linac) developed by LinaTech (LinaTech LLC, Sunnyvale, CA), a medical company. Recently, VenusX has been approved by the China Food and Drug Administration and is being used in clinics.^21^ Currently, VenusX is used for radiotherapy for various types of cancer, including brain tumors,^22^ lung cancer, and cervical cancer. A key innovation of VenusX is its orthogonal dual‐layer alpha multileaf collimator (αMLC), which consists of two layers of MLC positioned perpendicular to each other. The upper and lower orthogonal MLC cooperate to form the required field shape.

There are no published descriptions of the novel VenusX orthogonal dual‐layer αMLC. Such descriptions allow for a deeper understanding of its characteristics while providing quality assurance (QA) for the use of αMLC during radiotherapy. Therefore, this study aimed to provide a detailed description of the performance characteristics of αMLC.

## 2 METHODS

### 2.1 The VenusX medical linear accelerator and αMLC

**FIGURE 1:**
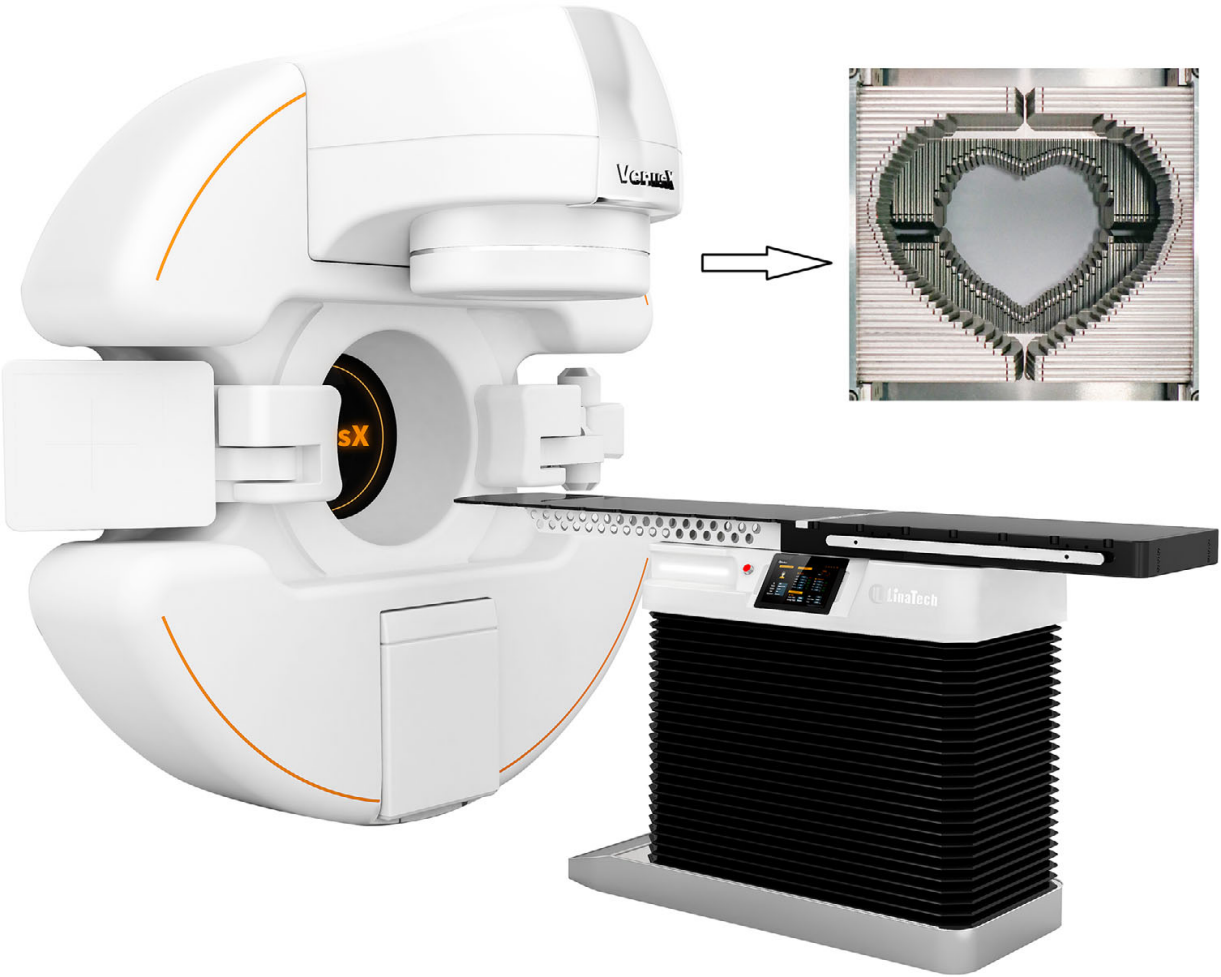
An illustration of the VenusX medical linear accelerator and αMLC. Physical diagram of αMLC from the patient's eye view. αMLC, alpha multileaf collimator.

**FIGURE 2:**
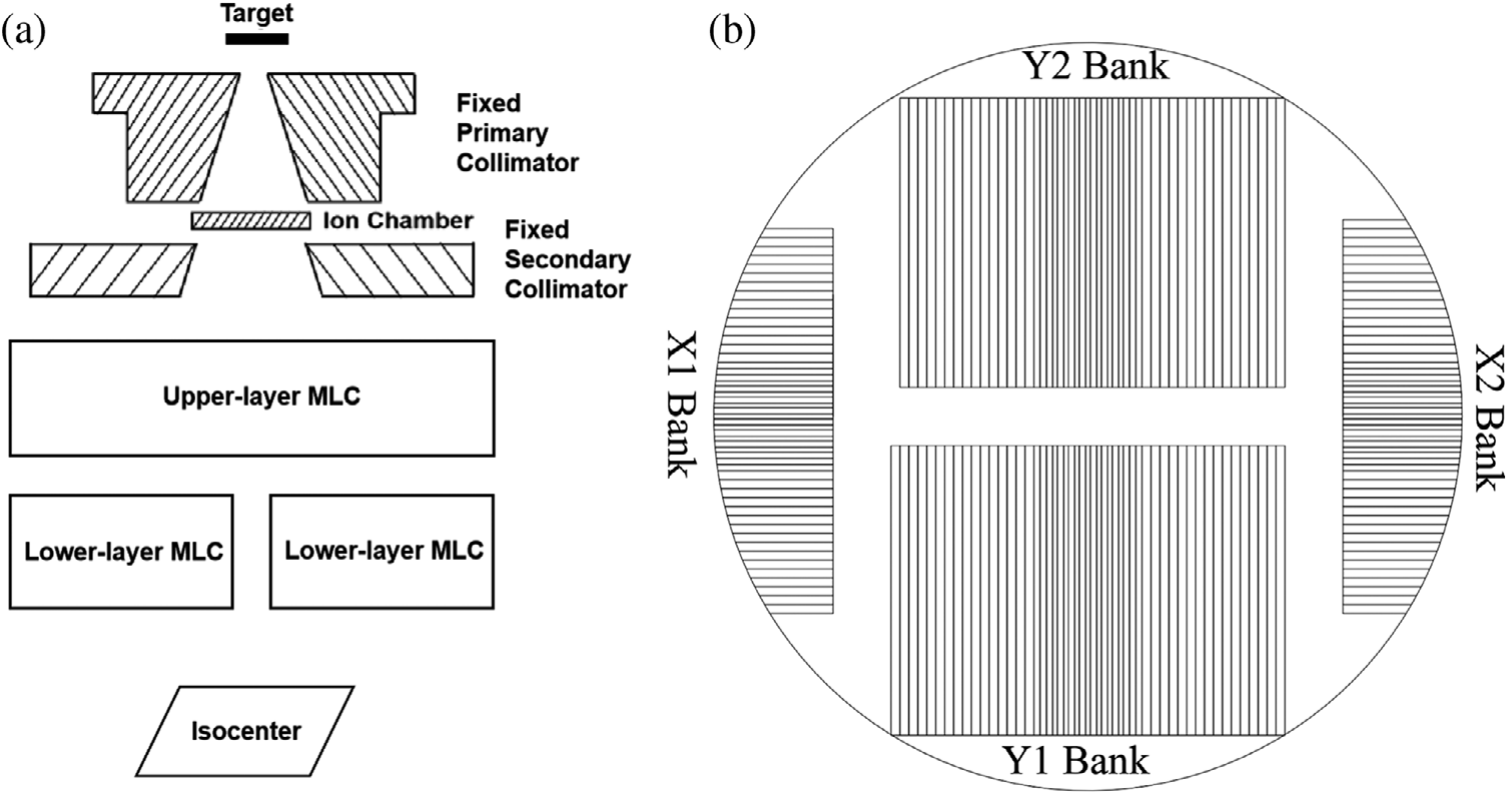
Schematic of the (a) VenusX head assembly and (b) two orthogonal layers of MLC banks. MLC, multileaf collimator.

The VenusX comprises a radiation treatment head, kilovoltage  and megavoltage imaging systems, a treatment couch, and other auxiliary systems. It can cover 3D conformal radiation therapy, IMRT, and VMAT treatment modes. The VenusX is equipped with kV‐CBCT and another mixed image‐guided mode. The VenusX linac uses 6 MV flattening filter‐free (FFF) x‐rays, and its source‐axis distance is 90 cm. Figure 1 depicts the physical attributes of VenusX and αMLC. Figure 2 shows a schematic of the VenusX head assembly and two orthogonal layers of MLC banks. In the current version of the VenusX linac, the collimator cannot be rotated. The primary and secondary collimators are fixed, not moving jaws.

The αMLC consists of two orthogonal layers of MLC machined using tungsten. Both the upper and lower layers consist of two banks with 51 pairs of leaves. The distance between the source and the upper layer is 24.7 cm, whereas that between the source and the lower layer is 36.4 cm. There are three physical thickness values for the leaves: 1.6, 2.2, and 3.1 mm. The architecture of the upper‐ and lower‐layer MLCs is identical. The physical thickness of the outermost 32 pairs (16 pairs on each side) of leaves is 3.1 mm. The middle 14 pairs of leaves are cross‐distributed with leaves of 1.6 and 2.2 mm physical thickness. The physical thickness of the remaining leaves is 2.2 mm. For the lower layer of the αMLC, the projection widths of the three leaf physical thicknesses at the isocenter were 4.0, 5.4, and 7.7 mm, respectively, whereas for the upper layer leaves, the projection widths were 5.9, 8.0, and 11.3 mm, respectively. The upper‐layer MLC moves along the Y‐axis (parallel to the gantry's rotation axis), whereas the lower‐layer MLC moves along the X‐axis (perpendicular to the gantry's rotation axis). The αMLC leaves are 7 cm in physical height, and each leaf is capable of over‐traveling to the full size of 40 or 20 cm from the center axis. Additionally, the αMLC has rounded leaf ends with a tongue and groove design allowing for interdigitation. The maximum leaf speeds of the upper and lower layers are 7 and 6 cm/s, respectively.

### 2.2 Electronic portal imaging device

The electronic portal imaging device (EPID) used in this study is a component installed on the VenusX accelerator. It consists of an image detection unit, image acquisition unit, image analysis software, and a dedicated workstation. The image detection unit mainly includes amorphous silicon detectors and related electronic circuits. Its effective detection pixel count is 2816 × 2816, with an actual pixel size is 0.15 mm, covering an effective detection area of 43 cm × 43 cm. When projected at the isocenter, its effective detection area reduces to 26 cm × 26 cm. The image acquisition unit comprises the drive and the image detection unit's acquisition electronics. EPID calibration involves several subcalibrations: dark field calibration, gain correction, pixel correction, correction of EPID sag during gantry rotation, and dose normalization calibration. The dark field serves as the background and is subtracted after EPID calibration. Dosimetric linearity and pixel size were verified after EPID calibration.^23^

### 2.3 Leaf positioning accuracy and reproducibility

We measured the positioning accuracy and reproducibility of the upper and lower layers of the αMLC, respectively. An automated MLC QA software was used to evaluate leaf positioning accuracy and reproducibility. This software was integrated into the VenusX linac and provided by LinaTech Medical Company for daily MLC QA. The picket fence test was conducted using the MLC QA software, and EPID was used to acquire images. The picket fence pattern comprised seven fields with a gap width of 2 cm at various positions. One layer of αMLC was opened to the maximum position, and the other layer was moved to different positions according to the set position. EPID was used to acquire images for each field. Leaf positioning accuracy was calculated by determining the deviation between the actual position and the nominal set position. Three independent repeat exposures were acquired on a working day as part of daily QA, and measurement data recorded from June 2020 to June 2021 were analyzed. Short‐term reproducibility was calculated by three independent repeat exposures on a working day, whereas long‐term reproducibility was determined as the average of the short‐term reproducibility values. To assess the impact of gravity on MLC leaf position accuracy, the same picket fence tests were also performed at gantry angles of 90°, 180°, and 270°, with a collimator angle of 0°.

### 2.4 Leaf transmission

#### 2.4.1 Average leaf transmission

Average leaf transmission was measured using a Farmer ionization chamber (IBA, FC65‐G) in solid water at a source‐to‐surface distance (SSD) of 90 cm. The ionization chamber was positioned along the beam central axis. The depth of dose maximum (*d*_max_) and 10 cm were set as the measurement depths with the beam dose set to 1600 MU. The depth of 10 cm was selected to ensure measurement stability. We evaluated the average leaf transmission separately for each leaf bank of single and dual αMLC layers.

To evaluate the average leaf transmission of the lower‐layer MLC, we first measured the dose when the X1 leaves of the lower‐layer MLC were fully closed. Then, we measured the dose when the X2 leaves of the lower‐layer MLC were fully closed. The average of all doses was calculated and normalized to the dose measured on the central axis of an open 10 cm × 10 cm field at the same depth. The same process was used to measure the average leaf transmission of the upper‐layer MLC.

To measure the average leaf transmission of both αMLC layers, doses were measured under fields that were fully blocked using the X1 leaves of the lower layer and the Y1 leaves of the upper layer, as well as the X2 leaves of the lower layer and the Y2 leaves of the upper layer. The same calculation and normalization method were used as for each single layer.

#### 2.4.2 Inter‐ and intraleaf transmission and leaf tip transmission

Leaf transmission consists of inter‐ and intraleaf transmission. We assessed the leaf transmission of the lower‐ and dual‐layer leaves using a Blue water phantom (IBA Dosimetry GmbH, Germany) with a CC13 chamber (IBA Dosimetry GmbH, Germany). Measurements were taken at a source‐to‐chamber distance of 90 cm at *d*_max_ in water. To assess the leaf transmission of the lower‐layer αMLC, the lower‐layer leaves were closed at an X position of −150 mm, whereas the upper‐layer leaves were opened. To assess the leaf transmission of the dual‐layer αMLC, the lower‐layer leaves were closed at an X position of −150 mm, whereas the upper‐layer leaves were closed at a Y position of +150 mm. The profiles were measured across the lower‐layer leaf travel direction at X positions of 0, +50, and +100 mm with a resolution of 1 mm. All measurements were normalized to the dose measured on the central axis of an open 10 cm × 10 cm field at *d*_max_.

To measure the leaf tip transmission of the lower‐layer αMLC, the lower‐layer leaves were closed at an X position of −100 mm, whereas the upper‐layer leaves were opened. To measure the leaf tip transmission of the dual‐layer αMLC, the lower‐layer leaves were closed at an X position of −100 mm, whereas the upper‐layer leaves were closed at a Y position of +100 mm. The profiles were taken at Y positions of 0 mm.

### 2.5 Leaf penumbra

We used Gafchromic EBT‐XD film (Ashland Inc., Bridgewater, NJ, USA) and solid water to measure the square and rhombus field penumbras formed by αMLC. The beam size of the square field was 10 cm × 10 cm. Two rhombus fields were used, with side lengths of 5 and 10 cm, respectively. The films were positioned in 10 cm of solid water with a backscatter of 5 cm and a source‐to‐axis distance of 90 cm. Because of the unflattened beam on VenusX, we calculated the penumbras using the method proposed by Ponisch et al.^24^ and Lim et al.^25^ Ponisch et al.^24^ introduced the concept of the inflection point for analyzing leaf penumbra of FFF beam. The maximum of the first derivative was calculated as the inflection point in the profile.^25^ Then, the profiles were normalized to the inflection point that corresponded to the 50% dose point. We calculated the MLC penumbra by determining the distance between the 80% and 20% dose points.

### 2.6 Dosimetric leaf gap measurement

Dosimetric leaf gap (DLG) was measured to account for the effect of rounded leaf ends. A Farmer ionization chamber was used for DLG measurements of the lower‐ and upper‐layer of the αMLC, performed at an SSD of 90 cm in solid water at a depth of 10 cm. The method followed the guidelines provided by Varian vendors.^26^ Charge readings were collected for sweeping MLC gap widths (2, 4, 6, 10, 14, 16, and 20 mm) spanning 120 mm at a constant speed. After accounting for the average MLC leaf transmission, a plot of the corrected gap reading versus the gap size was generated. A linear fit was applied, and the Y‐intercept provided the leaf rounded effect. The absolute value of the Y‐intercept represents the DLG.

### 2.7 MLC performance in field shaping

**FIGURE 3:**
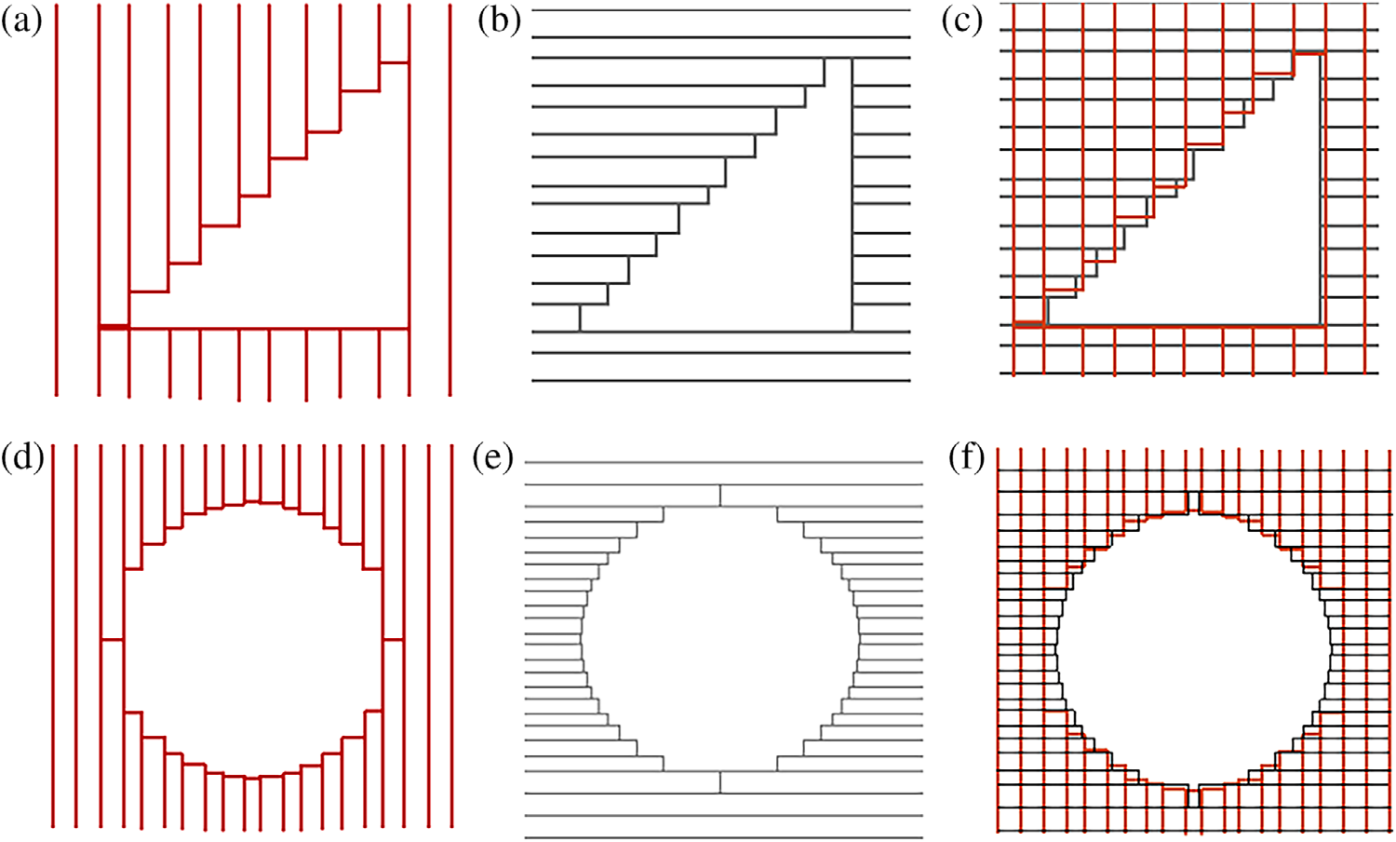
Schematic of right triangular and circular fields shaping by αMLC. (a) BEV of the right triangular field shaped by upper‐layer leaves of αMLC. (b) BEV of the right triangular field shaped by lower‐layer leaves of αMLC. (c) BEV of the right triangular field shaped by dual‐layer leaves of αMLC. (d) BEV of the circular field shaped by upper‐layer leaves of αMLC. (e) BEV of the circular field shaped by lower‐layer leaves of αMLC. (f) BEV of the circular field shaped by dual‐layer leaves of αMLC. BEV, beam‐eye view; αMLC, alpha multileaf collimator.

Right triangular and circular pattern fields were used to demonstrate the field‐shaping performance of the αMLC, as described by Liu et al.^18^ We used the right triangular field to investigate dose undulation at the field edge using αMLC, as shown in Figure 3a–c. A 10‐cm circular field was used to investigate the MLC dependence on the leaf stepping angle with αMLC, as depicted in Figure 3d–f. For single‐layer MLC, the stepping angle was determined as the angle between the direction of MLC movement and the tangent direction of the field edge curve. for dual‐layer MLC, the stepping angle was determined as the larger of the two stepping angles of the upper and lower layers.^18^ We compared the two evaluation pattern fields formed using both layers of the αMLC to corresponding fields formed using only a single layer of the αMLC. EPID was used for acquiring images.

## 3 RESULTS

### 3.1 Leaf positioning accuracy and reproducibility

**FIGURE 4:**
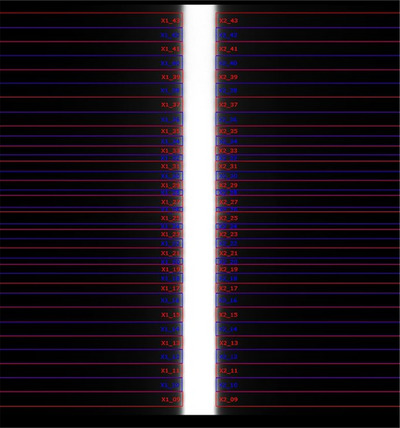
One of the picket fence fields captured by EPID. EPID, electronic portal imaging device.

The positioning accuracy and reproducibility were analyzed by grouping the leaves according to their respective layers. Figure 4 depicts one of the picket fence fields captured by EPID. The average maximum deviation between the actual arrival position of leaves and the target position was analyzed from June 2020 to June 2021. This value was 0.76 ± 0.09 mm for the upper‐layer leaves and 0.62 ± 0.07 mm for the lower‐layer leaves. The short‐term reproducibility of each layer was within 0.3 mm. The long‐term reproducibility of the upper‐layer leaves was 0.29 ± 0.03 mm, and that of the lower‐layer leaves was 0.26 ± 0.08 mm.

Table 1 shows one measurement of the leaf positioning deviation for the upper‐ and lower‐layer αMLC at the different gantry angles. The leaf positioning deviation at 90° and 270° gantry angles was less than 1 mm, but greater than at 0° and 180°. The maximum leaf positioning deviation was 0.90 mm for upper‐layer αMLC at a gantry angle of 90°.

**TABLE 1:** A measurement of the leaf positioning deviation for the upper‐ and lower‐layer αMLC at various gantry angles.

	Upper layer				Lower layer			
	0°	90°	180°	270°	0°	90°	180°	270°
Minimum deviation (mm)	0.1	0.2	0.1	0.2	0.1	0.2	0.1	0.2
Maximum deviation (mm)	0.7	0.9	0.7	0.8	0.6	0.8	0.7	0.8
Average deviation (mm)	0.5	0.7	0.5	0.6	0.4	0.6	0.5	0.6

### 3.2 Leaf transmission

#### 3.2.1 Average leaf transmission

Table 2 shows the average leaf transmissions for the upper‐, lower‐, and dual‐layer of the αMLC at the depth of *d*_max_ and 10 cm. The values at both depths were similar for the upper‐, lower‐, and dual‐layer of the αMLC.

**TABLE 2:** Average leaf transmission for each αMLC layer, and both αMLC layers.

	*d*_max_	*d* = 10 cm
Upper‐layer Y1	1.85%	1.86%
Upper‐layer Y2	1.87%	1.87%
Mean upper‐layer	1.86%	1.87%
Lower‐layer X1	1.81%	1.82%
Lower‐layer X2	1.82%	1.84%
Mean lower‐layer	1.82%	1.83%
Dual‐layer X1 and Y1	0.03%	0.03%
Dual‐layer X2 and Y2	0.03%	0.03%
Mean dual‐layer	0.03%	0.03%

#### 3.2.2 Inter‐ and intraleaf transmission and leaf tip transmission

**FIGURE 5:**
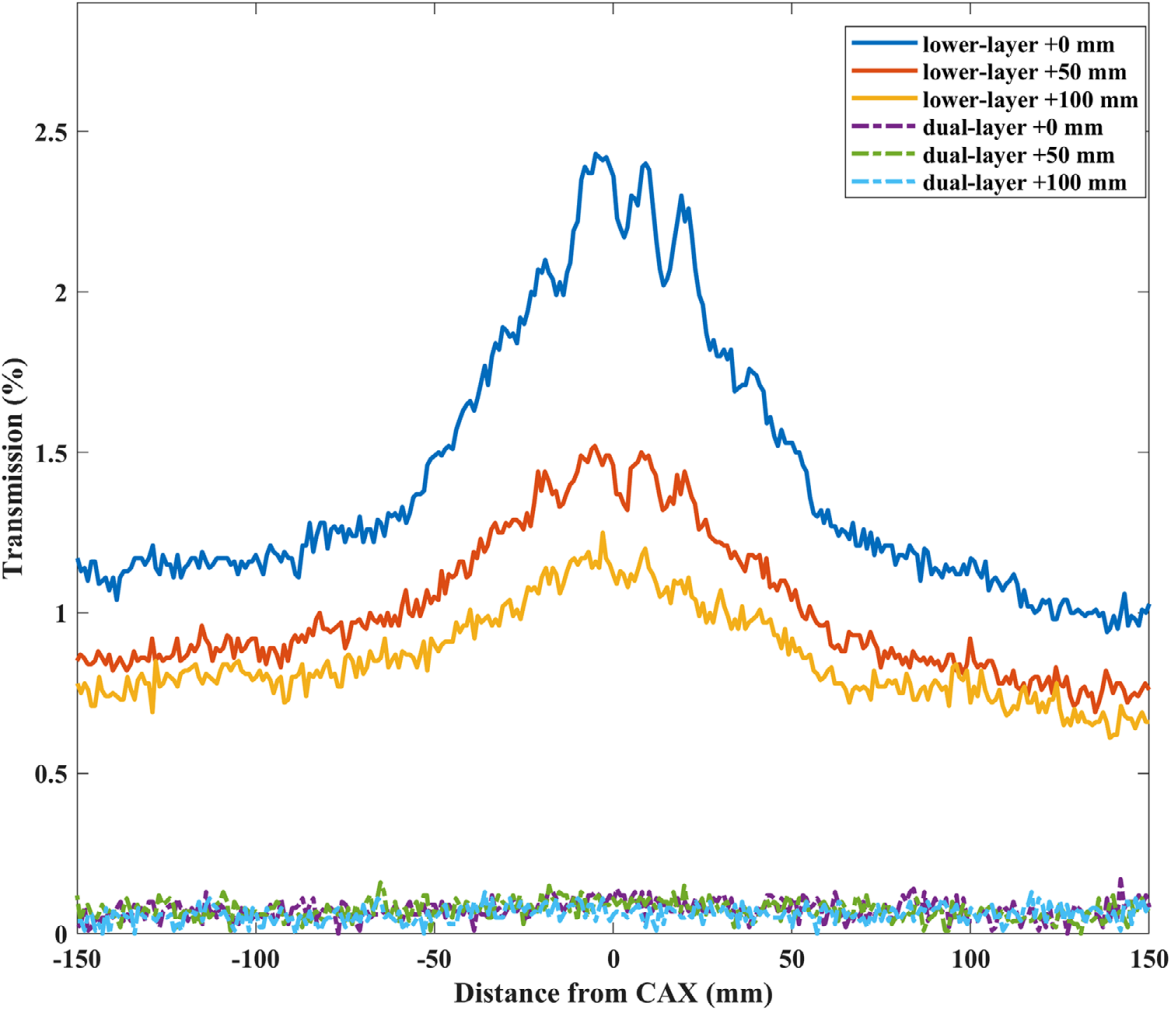
Lower‐layer and dual‐layer αMLC interleaf transmission profiles taken perpendicular to the direction of lower‐layer leaf travel at X positions of +0, +50, and +100 mm. αMLC, alpha multileaf collimator.

Figure 5 shows the inter‐ and intraleaf transmission profiles of the lower‐ and dual‐layer of the αMLC. The graphs depict profiles taken where the transmission was directly through the leaves and through the interleaf gaps. The maximum interleaf transmission was 2.43% of the lower‐layer leaves and 0.17% for the dual‐layer leaves.

**FIGURE 6:**
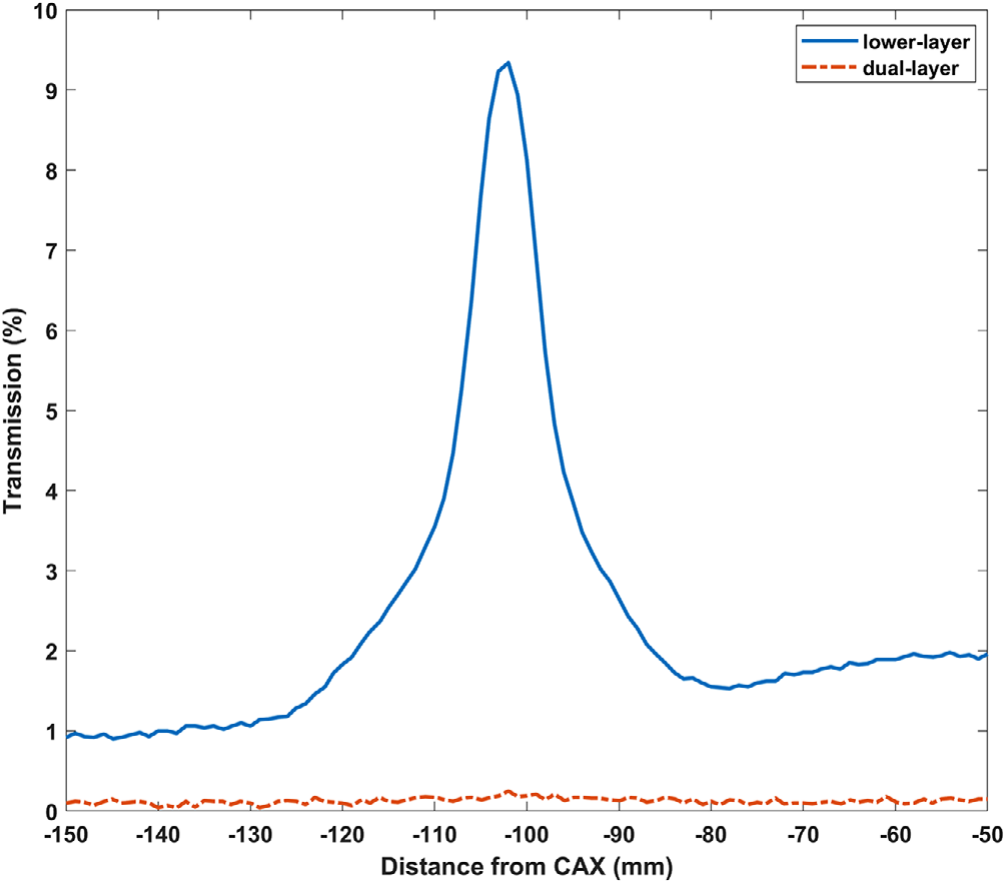
Lower‐layer and dual‐layer αMLC leaf tip transmission profiles taken parallel to the direction of lower‐layer leaf travel at a Y position of 0 mm. αMLC, alpha multileaf collimator.

Leaf tip transmission profiles for the lower‐ and dual‐layer leaves are depicted in Figure 6. The leaf tip transmission was 9.34% for the lower‐layer leaves and 0.25% for the dual‐layer leaves.

### 3.3 Leaf penumbra

**FIGURE 7:**
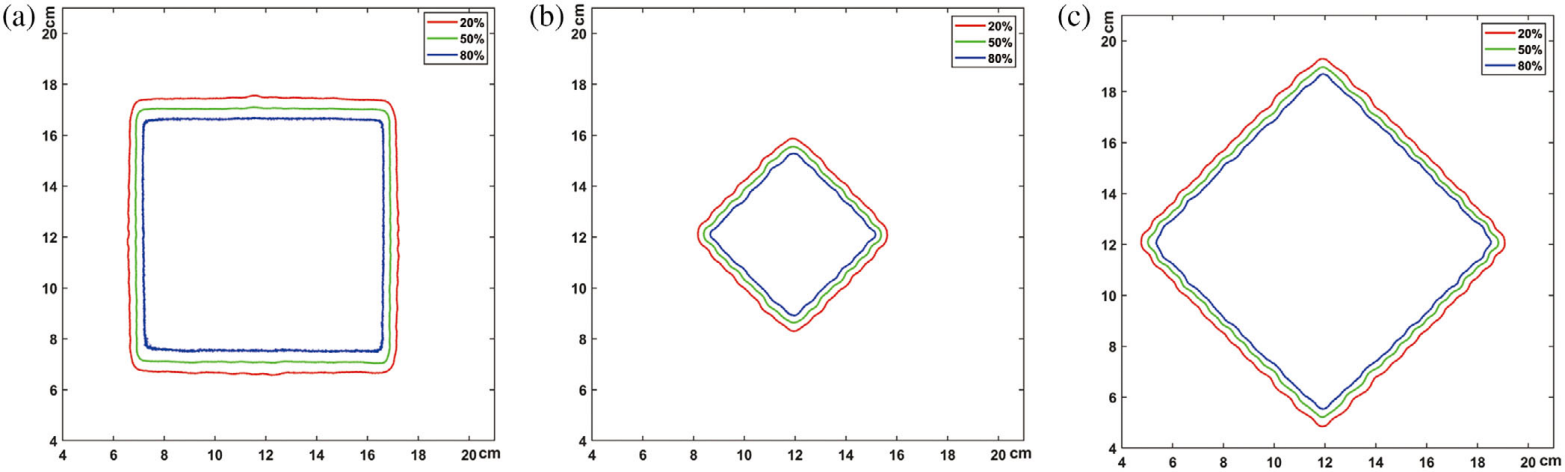
Isodose lines of square and rhombus fields shaped by dual‐layer leaves of αMLC. Films were used for image acquisition, and they were positioned at a depth of 10 cm in solid water with an SSD of 80 cm. (a) 10 cm × 10 cm square field; (b) 5 cm × 5 cm square field; (c) 10 cm × 10 cm square field. SSD, source‐to‐surface distance; αMLC, alpha multileaf collimator.

Figure 7 illustrates the isodose lines of square and rhombus fields shaped by dual‐layer leaves of αMLC. Table 3 presents the leaf penumbra of the different fields measured at a depth of 10 cm on VenusX. Leaf penumbra was measured in both the X and Y directions. The average leaf penumbra of the 10 cm × 10 cm square field was 6.2 mm in the X direction and 8.0 mm in the Y direction. The leaf penumbra of the rhombus field was smaller than that of the square field, with average penumbras of 3.6 and 4.9 mm for the rhombus fields with side lengths of 5 cm × 5 cm and 10 cm × 10 cm were, respectively.

**TABLE 3:** Film results of the penumbra for different fields of the αMLC measured at a depth of 10 cm in solid water (mm).

	X1	X2	Y1	Y2
Square field (10 cm × 10 cm)	6.2	6.1	8.2	7.8
Rhombus field (5 cm × 5 cm)	3.3	3.4	4.1	3.6
Rhombus field (10 cm × 10 cm)	5.3	5	4.8	4.3

### 3.4 DLG measurements

**FIGURE 8:**
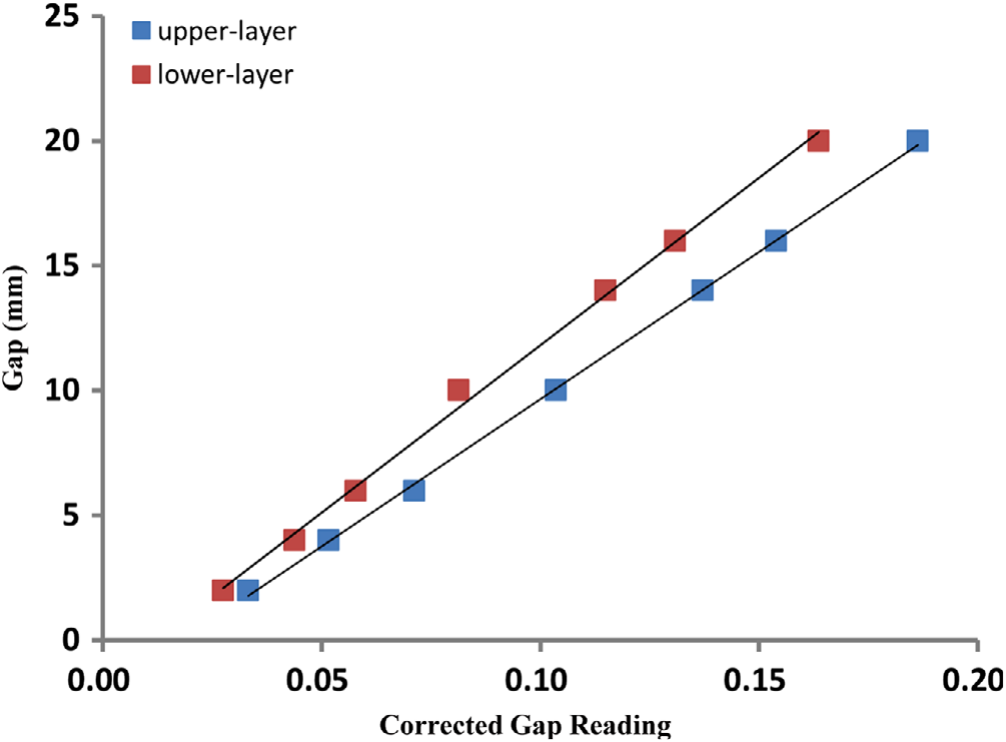
A plot of corrected gap reading versus the gap size of the lower‐ and upper‐layer αMLC. A linear fit was applied for the DLG measurement. DLG, dosimetric leaf gap; αMLC, alpha multileaf collimator.

Figure 8 shows the transmission‐corrected chamber readings for various gap widths and the linear fit line of the lower‐ and upper‐layer leaves. The measured DLGs of the lower‐ and upper‐layer leaves were 1.58 and 2.15 mm, respectively.

### 3.5 MLC performance in field shaping

**FIGURE 9:**
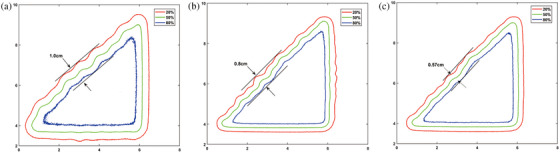
Isodose lines of right triangular fields separately shaped by upper, lower, and both layers of the αMLC. (a) Upper layer of the αMLC; (b) lower‐layer of the αMLC; (c) both layers of the αMLC. αMLC, alpha multileaf collimator.

**FIGURE 10:**
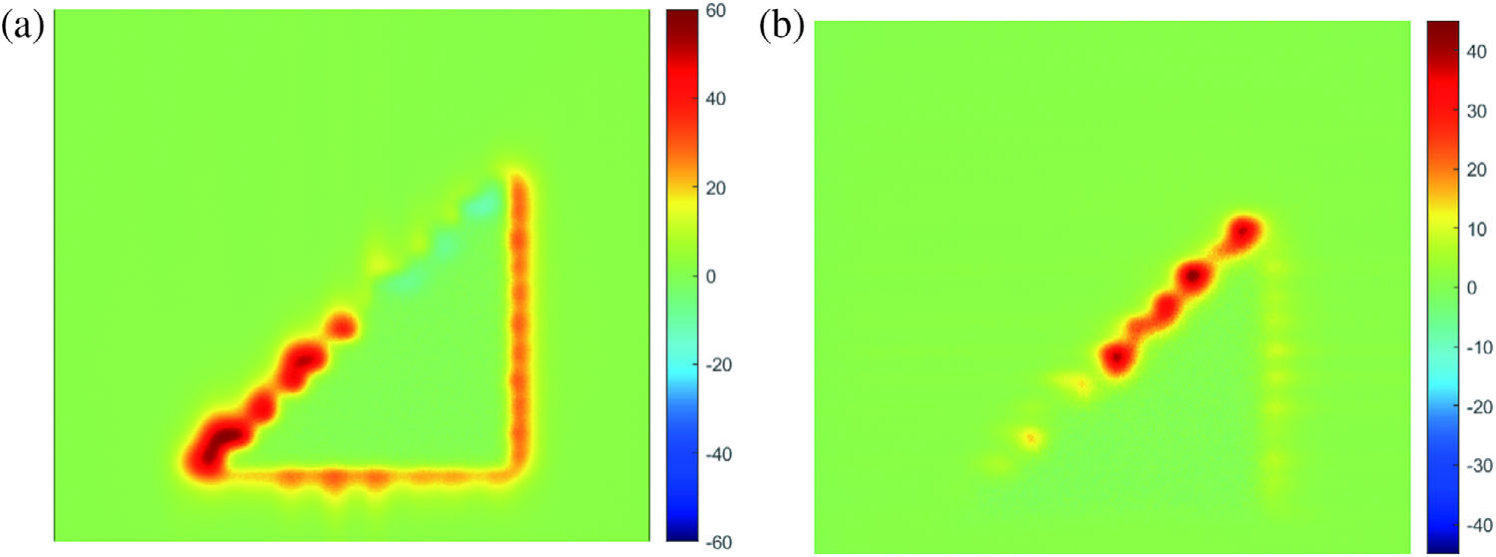
Planar dose comparison of the single layer αMLC subtracted by both layers of the αMLC. (a) Upper layer of the αMLC against both layers of the αMLC. (b) Lower layer of the αMLC against both layers of the αMLC. αMLC, alpha multileaf collimator.

Figure 9 presents the right triangular fields separately shaped by the upper, lower, and both layers of αMLC, as acquired by EPID at the corresponding isodose lines. As clearly shown in Figure 9, the right triangular field shaped by the dual‐layers leaves more closely matched the set field than that shaped by the single‐layer leaves. The dose undulation amplitudes of the 50% isodose line were 2.4, 2.2, and 0.6 mm for the upper, lower, and both layers, respectively. The dose undulation amplitude of the 50% isodose lines for the dual‐layer leaves was smaller than that for the single‐layer leaves. Figure 10 shows the results of planar dose subtraction for upper‐layer, lower‐layer again both layers of the αMLC. The percentage of normalized planar dose subtraction is indicated by the scale bar in Figure 10. The planar dose difference was primarily observed at the field edge.

**FIGURE 11:**
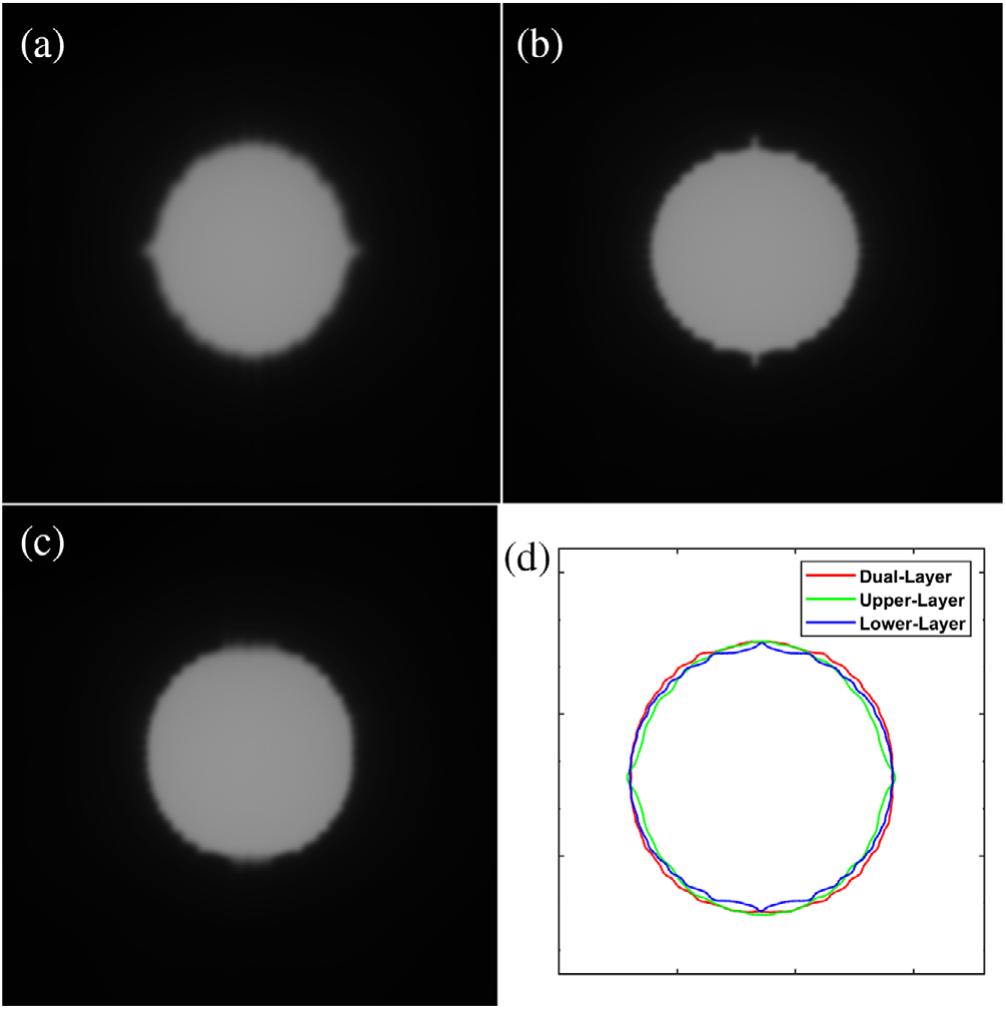
EPID image of the circular field formed by single‐ and dual‐layer αMLC and the corresponding 50% dose lines. (a) EPID image of the field formed by the upper layer of the αMLC; (b) field formed by the lower layer; (c) field formed by both layers; (d) 50% dose line corresponding to the three images. EPID, electronic portal imaging device; αMLC, alpha multileaf collimator.

Figure 11 shows the circular field images acquired using the EPID, along with the corresponding 50% isodose lines. Both layers of the αMLC shaped the circular field more closely matched the set field than that shaped by the single layer of the αMLC. The circular field formed by the upper‐layer MLC exhibited a stepping angle of 80.7° at the top and bottom of the circle, and 18.3° on the left and right sides. For the lower‐layer αMLC, the stepping angle of the circular field formed was 24° on the top and bottom sides and 82.9° on the left and right sides. The circular field formed by the dual‐layer αMLC had a stepping angle of 80.8° in the top and bottom directions and 82.9° in the left and right directions. The change in stepping angle for the dual‐layer αMLC on each side was smaller than that for the single‐layer αMLC. Figure 11 illustrates the benefit of the dual‐layer of the αMLC in reducing dependence on the leaf stepping angle.

## 4 DISCUSSION

Current MLCs typically feature a single layer^19^; however, multilayer MLCs have been gradually introduced to clinical settings.^25^, ^27^ Multilayer MLCs offer several important advantages, such as reducing normal organ dose outside the target volume, and requiring less time and fewer monitor units.^10^, ^19^, ^28^, ^29^, ^30^ Although dual‐layer micro‐MLC (DmMLC) uses an orthogonal dual‐layer design, there are two main differences between the DmMLC and αMLC. First, DmMLC is an add‐on MLC. Second, DmMLC is a micro‐MLC with a maximum possible field size of only 9.7 cm × 10.8 cm.^18^ The Halcyon has a dual‐layer MLC system, but its MLC uses stacked and staggered architecture. To the best of our knowledge, αMLC is the first orthogonal dual‐layer MLC integrated into the gantry's head. The MLC design and characteristics of the αMLC were reviewed for the first time in this study.

Leaf positioning accuracy affects treatment plan delivery, as inaccurate leaf positioning result in substantial dose deviations.^17^ We found that the leaf positioning accuracy and reproducibility of the αMLC were <1 mm, meeting the requirements outlined in the American Associate of Physicists in Medicine (AAPM) Task Group (TG) 142^31^ and TG 198^32^ reports, indicating acceptable αMLC stability.

MLC transmission is an important systematic parameter for treatment planning, especially for a more complex MLC leaf motions.^33^ Low‐dose leakage minimizes the risk of adverse effects by avoiding exposure to organs‐at‐risk.^34^ Multilayer MLCs feature lower dose leakage than single‐layer MLCs.^19^ The perpendicular placement of the upper and lower MLC layers can reduce intra‐ and interleaf transmissions. According to AAPM TG‐50, the average leaf transmission should be <2%,^35^ and the average single‐layer leaf transmission of the αMLC determined in the present study meets this requirement. The dual‐layer leaf transmission of the αMLC was also found to be significantly reduced compared to its single‐layer counterpart. The Elekta Agility MLC,^6^ Varian Millennium 120‐leaf MLC,^14^, ^15^ Cobalt‐60‐based ViewRay MRIdian MLC,^7^ and Euromechanics PMLC^17^ have measured average leaf transmission values of 0.35%, 1.36%, 0.153%, and 1.79%, respectively. The maximum leaf transmission values of these MLC systems are 0.44%, 1.8%, 0.35%, and 4.1%, respectively. Therefore, the dual‐layer αMLC demonstrates lower leaf transmission than the aforementioned commercial MLC systems.

We conducted separate DLG measurements for the lower and upper layers of the αMLC. Determining DLG for the orthogonal dual‐layer MLC was challenging due to the lack of a reference method. As the upper and lower layers of the αMLC can work together to define the edge of the field, the DLG of the dual‐layer MLC is minimal. We aim to address this issue in future research.

The dual‐layer αMLC more accurately approximated the right triangular and circular fields compared to the single‐layer MLC. The cooperation between the upper and lower αMLC layers enables precise shaping of the edge of the field. Using a dual‐layer αMLC can reduce dose undulation at the field's edge and reduce the MLC field's dependence on the leaf stepping angle. This results in improved performance characteristics for dose smoothing. Overall, the αMLC provides more precise field‐shaping at the field edges than the single‐layer MLC.

The design of the αMLC presents benefits in conformal radiotherapy treatments, particularly in cases such as the treatment of Hodgkin's lymphoma using mantle fields. With a substantial proportion of radiotherapy now relying on IMRT or VMAT, the clinical application of αMLC is still in its early stages. Further investigation is needed to determine whether its orthogonal dual‐layer design confers significant clinical benefits, particularly in terms of protecting normal tissues or organs. Additionally, aside from its characteristics, factors such as the MLC's inverse dose optimization algorithm, dose calculation engine, and beam delivery technique are closely related to the quality of IMRT or VMAT treatment plans.^10^ Our focus in this study was solely on describing the αMLC, overlooking other technical parameters associated with this novel orthogonal dual‐layer. Future studies will address the evaluation of the new optimization algorithm and beam delivery technique based on the orthogonal dual‐layer MLC to fully explore its advantages.

## 5 CONCLUSION

We systematically evaluated the performance characteristics of LinaTech's orthogonal dual‐layer MLC, the αMLC. The positioning accuracy and reproducibility of the αMLC were stable and in conformance with relevant standards. Owing to its dual‐layer design, transmission between and inside the leaves was remarkably reduced. As the upper and lower αMLC layers cooperate to define the field edges, the αMLC effectively smoothed the edges of the field. This reduction in dose undulation at the field's edge and the decrease in MLC field dependence on the stepping angle demonstrate the advantages of the dual‐layer αMLC.

## AUTHOR CONTRIBUTIONS

Qingxin Wang, Qifeng Li, and Wei Wang conceived the experiments. Qingxin Wang wrote original draft. Zhongqiu Wang, Chengwen Yang, and Daguang Zhang collected the data. Jun Wang and Ping Wang analyzed the data. All authors have read and approved the final manuscript. All authors contributed to review and editing of the manuscript.

## CONFLICT OF INTEREST STATEMENT

The authors declare that they have no conflicts of interest.
